# A Reason to Rethink Groups: New Approach Links PCBs, Thyroid Disruption

**DOI:** 10.1289/ehp.115-a505a

**Published:** 2007-10

**Authors:** Julia R. Barrett

The production of polychlorinated biphenyls (PCBs), once widely used in electrical transformers, plastics, and other products, has been banned in the United States since the 1970s. Nevertheless, most Americans carry measurable levels of PCBs because the compounds persist in the environment and bioaccumulate. Epidemiologic studies have linked prenatal PCB exposure with impaired neurodevelopment in infants and young children, and in animal studies, prenatal exposure caused decreased levels of the thyroid hormone thyroxine (T_4_). Given that thyroid hormones are essential for proper neurodevelopment, disruption of the thyroid system may be a pathway by which PCBs cause damage. New research using a novel method of grouping the chemicals now provides additional support for PCB-related thyroid disruption **[*EHP* 115:1490–1496; Chevrier et al.]**.

PCBs include 209 congeners that vary based on the number and positions of chlorine atoms. These congeners have been grouped according to their potential mechanism of action (e.g., estrogenicity, antiestrogenicity, or microsomal enzyme induction). In the current study, researchers grouped congeners on the basis of their reported abilities to induce the enzymes UDP-GT, CYP1A, and CYP2B. UDP-GT has a role in T_4_ elimination, and compounds that induce CYP1A and CYP2A are also likely to induce UDP-GT.

Thirty-four PCB congeners, in addition to other environmental chemicals, were measured in blood samples drawn from 285 pregnant women through the Center for the Health Assessment of Mothers and Children of Salinas, a longitudinal birth cohort study in the Salinas Valley, California. The women provided information through interviews on sociodemographic variables; alcohol, tobacco, drug, and caffeine consumption; and agricultural work history. Routine screening conducted by heel-stick blood collection yielded data on the infants’ blood levels of thyroid-stimulating hormone (TSH), which is involved in the control of thyroid function.

All of the infants had TSH levels within the normal range. However, levels were significantly higher in relation to specific PCB congeners in their mothers’ blood. When the researchers considered total PCB levels and PCBs grouped by structure or dioxin-like activity, they found no association. There was, however, a significant positive association between TSH levels and PCBs grouped by their ability to induce UDP-GT, CYP1A, and CYP2B. The team found that TSH increased by 29% with each 10-fold increase of such PCBs.

The researchers recommend that future studies evaluate whether any changes in neurodevelopment are associated with these PCB-related alterations in thyroid hormone levels. Their results suggest that grouping PCBs on the basis of hypothesized mechanism of action, rather than summing all congeners, may be important in understanding adverse effects of PCB exposure.

